# Measuring implementation behaviour of menu guidelines in the childcare setting: confirmatory factor analysis of a theoretical domains framework questionnaire (TDFQ)

**DOI:** 10.1186/s12966-017-0499-6

**Published:** 2017-04-04

**Authors:** Kirsty Seward, Luke Wolfenden, John Wiggers, Meghan Finch, Rebecca Wyse, Christopher Oldmeadow, Justin Presseau, Tara Clinton-McHarg, Sze Lin Yoong

**Affiliations:** 1 Hunter New England Population Health, Wallsend, NSW 2287 Australia; 2 School of Medicine and Public Health, University of Newcastle, Callaghan, NSW 2308 Australia; 3 Priority Research Centre for Health Behaviour, The University of Newcastle, Callaghan, NSW 2308 Australia; 4 Hunter Medical Research Institute, Newcastle, NSW 2300 Australia; 5 Ottawa Hospital Research Institute, Ottawa, ON K1H 8L6 Canada; 6 School of Epidemiology, Public Health and Preventive Medicine, University of Ottawa, Ottawa, ON K1H 8L6 Canada

**Keywords:** Psychometric properties, Implementation, Childcare, Theoretical domains framework, Guidelines

## Abstract

**Background:**

While there are number of frameworks which focus on supporting the implementation of evidence based approaches, few psychometrically valid measures exist to assess constructs within these frameworks. This study aimed to develop and psychometrically assess a scale measuring each domain of the Theoretical Domains Framework for use in assessing the implementation of dietary guidelines within a non-health care setting (childcare services).

**Methods:**

A 75 item 14-domain Theoretical Domains Framework Questionnaire (TDFQ) was developed and administered via telephone interview to 202 centre based childcare service cooks who had a role in planning the service menu. Confirmatory factor analysis (CFA) was undertaken to assess the reliability, discriminant validity and goodness of fit of the 14-domain theoretical domain framework measure.

**Results:**

For the CFA, five iterative processes of adjustment were undertaken where 14 items were removed, resulting in a final measure consisting of 14 domains and 61 items. For the final measure: the Chi-Square goodness of fit statistic was 3447.19; the Standardized Root Mean Square Residual (SRMR) was 0.070; the Root Mean Square Error of Approximation (RMSEA) was 0.072; and the Comparative Fit Index (CFI) had a value of 0.78.

**Conclusions:**

While only one of the three indices support goodness of fit of the measurement model tested, a 14-domain model with 61 items showed good discriminant validity and internally consistent items. Future research should aim to assess the psychometric properties of the developed TDFQ in other community-based settings.

**Electronic supplementary material:**

The online version of this article (doi:10.1186/s12966-017-0499-6) contains supplementary material, which is available to authorized users.

## Background

The World Health Organisation reports that in 2012 68% of deaths globally were attributed to non-communicable diseases [1]. Dietary patterns, food habits and food preferences developed in childhood track into adulthood and can impact on the onset of non-communicable disease [2]. Improving diet in children provides a critical opportunity to improve dietary behaviours in the overall population. Childcare is a particularly opportunistic setting in which to intervene to improve child dietary intake as it provides access to a large proportion of children at a key developmental period of their lives. Furthermore, children who attend centre-based childcare services consume up to 67% of their daily dietary intake while in care [3]. In Countries such as the United States (US) and the United Kingdom (UK) approximately one third of children attend some form of centre based childcare. During 2014, 54% of Australian children aged 2–3 years and 83% of Australian children aged 4–5 years attended centre based childcare [4].

Ensuring the availability of healthy foods to children through the implementation of menu dietary guidelines by childcare services is recommended as a means of improving the dietary intake of young children [5]. Evidence from systematic reviews suggests that interventions to improve food provision in childcare centres in line with dietary guidelines are effective in improving children’s diet [6, 7]. However, various studies have reported that the implementation of such guidelines by childcare services is sub-optimal [8–10]. For example a review of 118 nursery menus in the UK found that none complied with the menu dietary guidelines [9]. Similarly, in 2012, 46 menus from centre based childcare services in the Hunter New England region of New South Wales (NSW) Australia were audited and the study concluded that none complied with the menu dietary guidelines [8].

To improve the implementation of such guidelines to enhance child health, a greater understanding of implementation processes is required. While a number of tools have been used in the childcare setting to assess factors associated with implementation, the range of constructs assessed in previous studies has been limited, tending to focus on individual constructs such as staff knowledge and skills and attitudes [11–14]. The use of implementation theories or frameworks can be helpful in assessing the determinants of childcare provider behavior to ensure a broad range of factors that may impede or promote implementation are considered. Furthermore implementation frameworks can inform the selection of strategies to support implementation [15]. While such frameworks exist [16] few have comprehensive psychometrically evaluated measures to assess their constructs, limiting their utility.

One framework for which measures have been psychometrically evaluated is the Theoretical Domains Framework (TDF). The TDF is an implementation framework that aims to synthesize behavior change constructs that may affect (enable or impede) the implementation of evidenced based practices and guidelines [17]. The TDF was developed using a rigorous consensus and validation process, and represents an attempt to systematically summarize behavior change constructs covered in 33 theoretical models/frameworks. The initial version of the framework described 12 domains; however further construct validation research led to a 14 domain framework that is suggested to more comprehensively describe relevant behavior change constructs [17, 18].

There have been three previous studies that have empirically validated TDF constructs measures. These studies have been conducted with samples of university students and health care professionals. All three studies undertook a confirmatory factor analysis (CFA), a method that enables a researcher to test whether a hypothesised relationship exists between observed variables and their underlying constructs [19]. While the psychometric properties of these measures of TDF constructs differed across the three studies, most constructs showed reasonable fit to the model based on CFA [20–22]. These studies collectively conclude that methodological improvements are required to enhance the utility of the existing measures including ensuring that at least three items are available per construct and adding additional contextually relevant items applicable to the specific study setting and target audience.

Given the existing gaps in the literature regarding how to best support the implementation of dietary guidelines in the childcare setting, the importance of context in assessing determinants of implementation and the absence of validated tools which can be applied to professional practice regarding nutrition in the childcare setting this study aims to:

1. Develop and establish content validity for a 14-domain TDFQ that is contextually relevant for the childcare setting to measure the implementation behavior of sector specific menu dietary guidelines; and
2. Undertake a confirmatory factor analysis (CFA) and examine the psychometric properties of the developed measure (specifically reliability, discriminant validity and goodness of fit).

## Methods

### Design and setting

A cross-sectional survey was undertaken in centre based childcare services (specifically long day care services (LDCs)) in NSW, Australia. In Australia, LDCs are required to provide food which is consistent with the Australian Dietary Guidelines, as outlined in the dietary menu guidelines [23, 24].

Ethical approval to conduct the study was obtained from the Hunter New England Human Research Ethics Committee (reference 12/08/15/5.01) and the University of Newcastle Human Research Ethics Committee (reference H-2012-0321).

### Sample and recruitment

#### LDC services

A list of all LDC services in NSW (*n* = 2304) was provided to the research team by the NSW Ministry of Health. A random sample of 994 (43%) LDC services were selected by a statistician who had no further involvement in the study, using a random computer generator sequence. Of these, 342 (34%) LDC services met the eligibility criteria.

#### Service eligibility

LDC services were eligible to participate if the service: located within NSW Australia, was open for greater than 8 h per day; provided two mid-meals and one main-meal to children while in care; planned their menus onsite; prepared all their food onsite; and employed a service cook. LDC services that were externally catered for were excluded from the study. Services located in one region of NSW were also excluded, as they were participating in co-occurring research trials examining the implementation of nutrition-guidelines.

#### Service cook eligibility

Eligible service cooks had to be over 18 years of age, play a role in planning the service menu and be aware of the childcare specific dietary menu guidelines [24].

#### Recruitment

Service managers and cooks of the randomly selected services were mailed recruitment letters. The letters informed them of the study, explained how their study eligibility had been assessed, and invited the service cook to participate in a telephone interview. Approximately 1 week after the letter was sent, a telephone call was made to the service. Verbal consent to participate in the study was obtained from the service cook. If service cooks were unable to complete the survey at time of call an alternative time was scheduled. The service cooks were given the option to complete the survey across multiple calls. If the service employed more than one service cook, the cook who had the primary role in planning the service menu was asked to complete the interview.

### Procedure for the development of content for the TDFQ (content validity)

For this study the implementation behaviour of interest was guideline implementation. Two existing quantitative questionnaires which assessed constructs of the TDF (but had been previously developed for healthcare settings) were adapted for this study. Both of the existing TDF questionnaires were reported to have good construct validity and internal consistency [22, 25] and utilised the 14 domain structure, with domains being:1) Knowledge; 2) Skills; 3) Social/professional role and identity; 4) Beliefs about capabilities; 5) Optimism; 6) Beliefs about consequences; 7) Reinforcement; 8) Intentions; 9) Goals; 10) Memory, attention and decision processes; 11) Environmental context and resources; 12) Social influences; 13) Emotion; and 14) Behavioural regulation [18]. Definitions of the constructs these domains capture can be seen in Table 1.

**Table 1:** Definition of TDF constructs by domain, and as applied to the childcare setting and the implementation of menu dietary guidelines

Domain	Definition of constructs [18]	Application to the childcare setting
Knowledge	Knowledge (including knowledge of condition/scientific rationale), Procedural knowledge, Knowledge of task environment	The service cooks awareness and familiarity with implementing the menu dietary guidelines
Skills	Skills, Skills development, Competence, Ability, Interpersonal skills, Practice, Skill assessment, Coping strategies	Training, skills and practice in implementing the menu dietary guidelines
Professional role and identity	Professional identity, Professional role, Social identity, Professional boundaries, Professional confidence, Group identity, Leadership, Organisational commitment	The extent that implementation of menu dietary guidelines is perceived as part of the service cook’s role
Beliefs about capabilities	Self‐confidence, Perceived competence, Self‐efficacy, Perceived behavioural control, Beliefs, Self‐esteem, Empowerment, Professional confidence	The service cooks confidence in implementing the menu dietary guidelines
Optimism	Optimism, Pessimism, Unrealistic optimism, Identity	The service cooks confidence that the implementation of the menu dietary guidelines will be attained
Beliefs about consequences	Beliefs, Outcome expectancies, Characteristics of outcome expectancies, Anticipated regret, Consequents	The service cooks belief about benefits/disadvantages of implementing the menu dietary guidelines
Reinforcement	Rewards (proximal/distal, valued/not valued, probable/improbable), Incentives, Punishment, Consequents, Reinforcement, Contingencies, Sanctions	The extent of recognition and reward the service cooks expect to receive when implementing the menu dietary guidelines
Intentions	Stability of intentions, Stages of change model, Trans-theoretical model and stages of change	The service cooks intention to implement the menu dietary guidelines
Goals	Goals (distal/proximal), Goal priority, Goal/target setting, Goals (autonomous/controlled), Action planning (with relation to their intention to implement	The relative importance to service cooks of implementing the menu dietary guidelines
Memory, attention and decision processes	Memory, Attention, Attention control, Decision making, Cognitive overload/tiredness	The extent to which implementing the menu dietary guidelines is part of regular practice
Environmental context and resources	Environmental stressors, Resources/material resources, Organisational culture/climate, Salient events/critical incidents, Person x environment interaction, Barriers and facilitators	The environmental context/situation that may encourage/discourage implementation of the menu dietary guidelines
Social influences	Social pressure, Social norms, Group conformity, Social comparisons, Group norms, Social support, Power, Intergroup conflict, Alienation, Group identity, Modelling	The interpersonal relationships/process that may influence implementation of the menu dietary guidelines
Emotions	Fear, Anxiety, Affect, Stress, Depression, Positive/negative affect, Burn‐out	Service cooks emotions when implementing the menu dietary guidelines
Behavioural regulation	Self‐monitoring, Breaking habit, Action planning (with relation to monitoring their habits)	Service cooks ability to self-monitor and action plan to implement the menu dietary guidelines

Modification of the questionnaire items to be relevant to the LDC setting was overseen by the research team, which included: experienced health promotion practitioners; implementation scientists; psychologists; dietitians; psychometricians; and behavioral scientists. Modification was based on feedback from stakeholders and expert opinion. Changes to the questionnaire took into consideration individual factors and other inner setting implementation constructs, such as structural characteristics, networks and communication, and culture, which could impact on implementation behavior in the setting [26]. An example of how items were modified can be seen in Table 2.

**Table 2:** Example of a modified item

Original item	If I *[action]* in *[context, time]* with *[target population]* it will benefit public health
Modified item	I believe *[action related to program, intervention, innovation or guidelines]* according to the *[name of recommendations, protocol, guidelines]*, will lead to benefits for the *[participants, clients, patients, individuals, children]*
Item tailored for LDC setting^a^	I believe *planning a menu* according to the *Caring for Children guidelines,* will lead to benefits for the *children who attend the service*

^a^Full set of LDC items included in Additional file 1

All items were modified in a generic format (see Additional file 1), allowing for adaptation of the items to different programs, guidelines, interventions or initiatives that may be undertaken in other community settings. Context specific prompts were included, with six of the questionnaire items to ensure they were interpreted in relation to the LDC setting. Consistent with previous questionnaires, the final items required respondents to answer using a 7-point Likert scale (strongly agree to strongly disagree).

The survey was piloted with two LDC service cooks who were not included in the final sample. Piloting indicated that completion of the survey took 25 min; it was well understood and was positively received by the respondents. No amendments were made to the survey following the pilot.

### Data collection procedure

#### Quality assurances

The final questionnaire was administered to LDC service cooks via a computer assisted telephone interview (CATI) over a 3-months period. The telephone interview was scripted to ensure standardised delivery. All CATI interviewers were experienced in using standardised telephone interviewing protocols in the conduct of telephone based health surveys. All interviewers received 1-day training and each interviewer conducted one mock interview with a member of the research team prior to administering the questionnaire to respondents.

#### Characteristics of service cooks

The CATI interview included items to capture the demographic and professional characteristics’ of the service cooks (age, gender, education level, years employed as a service cook, weekly hours worked) and the current processes that related to the planning of menus in their service and the provision of healthy foods (how often they review or plan a service menu, whether they received support to plan the service menu, the cycle length of the menu).

### Data analyses

#### Reliability (prior to CFA)

The reliability (internal consistency) of the newly developed 14 domain TDFQ was assessed using Cronbach’s alpha (α), with an alpha between 0.70 and 0.95 considered acceptable [27].

#### CFA

To assess construct validity of the questionnaire, CFA was undertaken using the CALIS procedure of SAS. The measurement model hypothesised the relationship between the latent domains and manifest variables that measure these domains. For the CFA, based on previous publications a prior hypothesis was made that each item (*n* = 75) loaded on only one domain and that a number of domains were potentially correlated (i.e. knowledge and skills; social/professional role and skills) (See Appendix A) [22]. No directional paths between latent domains were hypothesised. All loadings were standardized (domain means and variances fixed at 0 and 1 respectively). The model was estimated using the full information maximum likelihood method, which used all available data. Questionnaire items worded negatively (*n* = 3) were reverse-coded for analysis.

The model was assessed for goodness of fit and iteratively revised in an attempt to derive a theoretically meaningful and statistically acceptable model. Items were dropped for the following conditions: 1) the item had a non-significant loading on its factor (*p*-value > 0.05); 2) the item appeared more than once in the ten highest Lagrange multiplier (LM) estimates on different factors (a high LM estimate indicates a complex item which should be removed); 3) the item had a loading <0.4 on its factor domain [28, 29].

As per recommendations, an attempt was made to include three items per domain to ensure minimum coverage of each construct’s theoretical domain.

The sensitivity of the model was assessed by restricting the number of domain co-variances to the following pre-specified domains which the TDF literature suggests are correlated: 1) Domain 1 (Knowledge) and Domain 2 (Skills); 2) Domain 3 (Social/professional role) and Domain 2 (Skills) [22].

When co-variances between the remaining domains were fixed to zero, the model fit was worse, suggesting that estimating all co-variances between the domains produced a better model.

#### Goodness of fit

As the chi-square test statistic is generally regarded as being too stringent, particularly when data is not multivariate normal, a chi-square to degrees of freedom ratio was reported, where a ratio of less than two was considered ideal [30]. Consistent with best practice recommendations, three goodness-of-fit measures were also reported: one absolute measure (Standardized Root Mean Square Residual (SRMR)), one parsimony index (Root Mean Square Error of Approximation (RMSEA)), and one incremental index (Comparative Fit Index (CFI)) [31]. An SRMR value less than 0.055 is considered ideal [31]. An RMSEA value <0.05 indicates a close approximate fit, while values between 0.05 and 0.08 suggest an acceptable fit and values >0.10 suggest a poor fit [32, 33]. A CFI value exceeding 0.9 indicates good fit [33].

#### Reliability (following CFA)

The reliability (internal consistency) of the revised TDFQ was assessed using Cronbach’s alpha (α), with an alpha between 0.7 and 0.95 considered acceptable [27].

#### Discriminant validity

Discriminant validity was assessed following the method proposed by Anderson and Girbing [19]. The estimated correlation parameter for two estimated constructs was constrained between them to 1.0 and a chi-square difference-test was performed on the values obtained for the constrained and unconstrained models. The test was performed with one pair of factors at a time, as a non-significant test for one pair of factors can be made unclear by being tested with several pairs that have significant values [19]. A further assessment of discriminant validity was undertaken which was to determine whether the confidence interval (+/− two standard errors) around the correlation estimate between the two factors included 1.0 (the confidence interval test). Intervals that did not include 1.0 were considered supportive of factor discriminant validity.

## Results

### Characteristics of service cooks

Of the 994 randomly sampled services, 342 (34%) met the eligibility criteria. Main reasons for services ineligibility included, the service did not provide two mid-meals and one main meal per day; and the service was externally catered therefore not all food was prepared on-site by a service cook. Of the 342 eligible services, 202 (59%) service cooks completed the full CATI which included demographic items and the 75 item TDFQ. One-hundred and forty (41%) service cooks did not complete all 75 items of the TDFQ as they were unaware of certain aspects of the sector specific menu dietary guidelines. There were significant differences in the characteristics of respondent and non-respondent service cooks for three variables; those who had competed a registered training organisation course; >5 years in current position and; <5 h taken to plan a new service menu (Table 3).

**Table 3:** Demographics of non-respondent service cooks

	Respondents *n* = 202	Non-respondents^a^ *n* = 140	*p*-value**
Demographic variable	*N* (%)	*N* (%)	
*Female*	188 (93.07)	127 (90)	0.43
Qualification			
*University Qualification*	4 (1.98)	2 (1.43)	0.70
*Tafe Course*	88 (43.56)	56 (40.00)	0.58
*Registered Training Organisation Course*	88 (43.56)	78 (55.71)	0.03
*‘On the job’ Training*	44 (21.78)	27 (19.29)	0.59
*≥40 years of age*	130 (64.68)	88 (62.86)	0.73
>5 years as a service cook in childcare services	109 (56.19)	66 (47.83)	0.15
>5 years in current position	83 (41.92)	40 (28.99)	0.02
Hours worked per week			0.89
*20*–*29 h*	82 (40.59)	60 (43.17)	
*Works ≥30 h per week*	93 (46.04)	61 (43.88)	
Menu planning practices			
Menu cycle Length			0.42
*Six week menu cycle*	31 (15.35)	12 (8.57)	
*Four week menu cycle*	108 (53.47)	78 (55.71)	
*Two week menu cycle*	14 (9.41)	11 (7.86)	
Frequency service plans a menu			0.92
*Every 6 months*	84 (41.58)	63 (45.00)	
*Every 3 months*	60 (29.70)	38 (27.14)	
Hours taken to plan a service menu			0.04
*< 5 h*	127 (62.87)	96 (68.57)	
*≥ 5 h*	66 (32.67)	31 (22.14)	

^a^Non-respondents are those eligible service cooks who did not complete all 75 TDFQ items and whose data is not included in the analysis

**p-value <0.05 is considered significant

Of the 202 service cooks who completed the full CATI, 93% were female, 56% had >5 years’ experience working as a service cook and 42% had been in their current position for >5 years. The most common menu cycle length was 6 weeks (53% of respondents). Services typically planned a new service menu every 6 months (42% of respondents) and 63% of respondents reported it took <5 h to plan a new service menu.

### Psychometric properties of the questionnaire

The final TDFQ consisted of 75 items. Each domain had a minimum of three items as per recommendations [34] The domains and items were: Knowledge (5 items); Skills (3 items); Social/professional role and identity (3 items); Beliefs about capabilities (6 items); Optimism (3 items); Beliefs about consequences (8 items); Reinforcement (4 items); Intentions (3 items); Goals (4 items); Memory, attention, and decision processes (4 items); Environmental context and resources (8 items); Social influences (5 items); Emotion (6 items); Behavioural regulation (8 items).

#### Reliability (prior to CFA)

The Cronbach’s Alphas for the 14 domains prior to CFA ranged between 0.56 and 0.90 and are reported in Table 4.

**Table 4:** Number of domain items and Cronbach Alpha’s for original and revised TDFQ

Domain	Original TDFQ (75 items)		Revised TDFQ (61 items)	
	# of items	α	# of items	α
Knowledge	5	0.85	5	0.85
Skills	3	0.61	3	0.61
Professional role and identity	3	0.88	3	0.89
Beliefs about capabilities	6	0.80	6	0.80
Optimism	3	0.67	3	0.67
Beliefs about consequences	8	0.71	4	0.89
Reinforcement	4	0.73	4	0.73
Intentions	4	0.90	4	0.90
Goals	4	0.68	4	0.67
Memory, attention and decision processes	4	0.56	3	0.64
Environmental context and resources	9	0.77	7	0.79
Social influences	6	0.64	4	0.68
Emotion	7	0.89	5	0.88
Behavioural regulation	9	0.76	6	0.80

#### CFA

Five iterative processes of adjustment were undertaken for the CFA. Across the five iterations one item was removed for non-significant loading on a construct, six items were removed for a high LM on different factors and seven items were removed for small loadings of <0.4.

One item (item 8) had high LM estimates on different factors but was not removed, as its removal would have resulted in a 2-item factor.

In total, 14 TDFQ items were removed, leaving 61 items (See Additional file 1). No questionnaire items moved between domains across the 5 iterations and none of the 14 domains were removed from the measure. Each domain resulted in having 3–6 items as identified by the CFA.

#### Goodness of fit

The Chi-Square goodness of fit statistic was 3447.19 (with 1678 degrees of freedom), providing a *p*-value of <0.0001. The Chi-Square statistic divided by the degrees of freedom was 2.5 (a ratio of less than 2 is considered ideal) leading to rejection of the null hypothesis (that the model fits the data).

The SRMR obtained for the final model was 0.070 suggesting the model was not ideal. The RMSEA and 90% confidence interval for this model was 0.072 (0.069, 0.076) suggesting that the model has reasonable fit on this criteria. The CFI had a value of 0.78, suggesting that the model did not fit the data well.

#### Reliability (following CFA)

For the final 61 items, Cronbach’s alphas ranged from 0.61 (skills) to 0.90 (intentions). Nine of the 14 domains demonstrated reliability (internal consistency) with alphas between 0.70 and 0.90. The remaining four domains had Cronbach’s alphas below 0.70 (Skills α = 0.61; Optimism α = 0.67; Goals α = 0.67; Social influences α = 0.68) (Table 3).

#### Discriminant validity

The confidence interval test showed that there were no correlations between factors that had 95% confidence intervals including 1.0, therefore supporting the discriminant validity of the model.

## Discussion

This is the first empirical validation of a TDFQ measure in the non-health care setting. The final 14-domain, 61-item measure demonstrated satisfactory fit on one of the three goodness-of-fit-tests, has internally consistent items, and overcomes some of the previous limitations of existing tools in the field. The questionnaire displayed good discriminant content validity, indicating that the domains measure theoretically different constructs. This result is congruent with previous TDF measures which also demonstrated good discriminant content validity [22, 25].

The two previous TDF measures developed by Taylor et al., based on the original 12 domain framework have demonstrated satisfactory fit, with one measure meeting both of the goodness of fit measures and the other meeting one of the two goodness of fit measures [20, 21]. Both 12 domain measures displayed a chi-square to degrees of freedom ratio less than 2.0 and an RMSEA score greater than 0.05 [20, 21]. When assessing the reliability of the TDFQ, the domain ‘Social influences’ performed poorly, however this aligns with previous research reported by Taylor et al., whom employed the same acceptable alpha cut-off point of 0.7 [21].

### Considerations for future development of the TDFQ

Tools that can provide a robust assessment of implementation constructs are important to help assess implementation barriers, guide the development of implementation strategies and to understand implementation mechanisms. In this study, two of the three goodness of fit statistics did not support the final model. Such findings, therefore, suggests that further development work of the tool is warranted.

#### Response scale

A large number of items had only two response options selected by participants (strongly agree and agree), suggesting a high ceiling effect. While we included a seven point response scale (recommended for improving validity) it is possible that the response options did not allow for sufficient diversity in respondent preferences [35]. The administration of the questionnaire via telephone interview, rather than paper and pencil, may have also impacted on the way in which participants responded to the questionnaire items. Further pilot testing of response scales should be conducted prior to administering the TDFQ.

#### Feasibility and acceptability

The feasibility (how long it takes to administer and score) and acceptability (ease of completion) of the TDFQ should be considered in future research to reduce the risks of the tool not being used. Further factor analysis should be performed to determine whether there is potential to reduce the number of items in this setting and other community-based settings.

#### Sample size

Given there were some minor revisions to the original hypothesised model, it is recommended that the fit of this measure be assessed with a larger samples of service cooks in the childcare setting, as well as with other staff providing menu services in similar community settings (schools, sporting clubs). When validating this measure, researchers should undertake a power calculation and aim to reach the appropriate sample size to allow for increased confidence in results. Thorough psychometric testing and reporting (reliability and validity) should be undertaken to increase the empirical evidence available for the use of a TDF measure for intervention development and evaluation of implementation strategies [36].

### Limitations

There are a few limitations to the current study. First, the majority of the respondents were female (93%). While this reflects the demographic for our population of interest (service cooks in childcare settings), this may affect the generalisability of the results to other community settings. Second, a significant proportion of service cooks who were telephoned and invited to participate had not heard of the sector-specific menu dietary guidelines (46%) and were not eligible to participate in this study. The findings of the current study only apply to cooks who were already aware of the guidelines, and as such may have higher intentions of applying such guidelines.

Third, the acceptability of the questionnaire was not specifically measured. However, the average time to complete the telephone interview was 25–40 min, which may represent a significant burden for respondents. This highlights the difficulties in developing a comprehensive questionnaire that satisfactorily measures the 14 TDF domains, while being of reasonable length [25]. Fourth, only a small sample size was available for the analysis, as only data for respondents who completed all 75 items of the TDFQ were included. The final sample size of 202 may have been inadequate to psychometrically validate the 75 item TDFQ as the ratio of “five participants per item” was not achieved (i.e. 375 participants were needed to meet this recommendation) [32].

## Conclusion

To our knowledge this is the first quantitative measure of the TDF developed for application in a non-health care setting, specifically the childcare setting. While only one of the three indices support goodness of fit of the measurement model tested, with some minor revisions we arrived at a 61-item model with good discriminant validity, with internally consistent items. Future research should aim to assess the psychometric properties of the developed TDFQ in the childcare setting and other community settings.

## Additional file

Additional file 1: Displays the generic format of the TDFQ and the final TDFQ items. Final 14-domian, 61-item theoretical domain framework questionnaire. (DOC 88 kb)

## Abbreviations

CFA: Confirmatory factor analysis
CFI: Comparative fit index
LDCs: Centre based long day care services
RMSEA: Root mean square error of approximation
SRMR: Standardized root mean square residual
TDF: Theoretical domains framework
TDFQ: Theoretical domains framework questionnaire
