# Supplementary material for: Measuring implementation behaviour of menu guidelines in the childcare setting: confirmatory factor analysis of a theoretical domains framework questionnaire (TDFQ)
Source: Int J Behav Nutr Phys Act. 2017 Apr 4;14:45. doi: 10.1186/s12966-017-0499-6 (PMC5381057; doi:10.1186/s12966-017-0499-6)
Supplement: Additional file 1: — Displays the generic format of the TDFQ and the final TDFQ items. Final 14-domian, 61-item theoretical domain framework questionnaire. (DOC 88 kb) [file 12966_2017_499_MOESM1_ESM.doc]

Additional File 1

File format: Final 14-domian, 61-item theoretical domain framework questionnaire.doc

Title: Final 14-domian, 61-item theoretical domain framework questionnaire

**Description of data: Displays the generic format of the TDFQ and the final TDFQ items. Final 14-domian, 61-item theoretical domain framework questionnaire**

| **Domain** | Item # | **Generic Structure of item** | **Item included in Cooks survey** |
| --- | --- | --- | --- |
| 1. Knowledge | 1. | **I am aware of the content of the** [insert name of recommendations, protocol, guidelines] | **I am aware of the content of the** Caring for Children guidelines |
|  | 2. | **I am aware of the objectives of the** [insert name of recommendations, protocol, guidelines] | **I am aware of the objectives of the** Caring for Children guidelines |
|  | 3. | **I know what my responsibilities are,** **with regard to** [insert action related to program, intervention, innovation or guidelines] **according to the** [insert name of recommendations, protocol, guidelines] | **I know what my responsibilities are,** **with regard to** planning a menu **according to the** Caring for Children guidelines |
|  | 4. | **I know how to** [insert action related to program, intervention, innovation or guidelines] **according to the** [insert name of recommendations, protocol, guidelines] | **I know how to** plan a menu **according to the** Caring for Children guidelines |
|  | 5. | **I know when to apply** [insert name of recommendations, protocol, guidelines] **when** [insert action related to program, intervention, innovation or guidelines] | **I know when to apply** the Caring for Children guidelines **when** planning a menu |
| 2. Skills | 6. | **I have received training regarding how to** [insert action related to program, intervention, innovation or guidelines] **according to the** [insert name of recommendations, protocol, guidelines] | **I have received training regarding how to** plan a menu **according to the** Caring for Children guidelines |
|  | 7. | **I have the skills needed to** [insert action related to program, intervention, innovation or guidelines] **according to the** [insert name of recommendations, protocol, guidelines] | **I have the skills needed to** plan a menu **according to the** Caring for Children guidelines |
|  | 8. | **I have been given the opportunity to practice** [insert action related to program, intervention, innovation or guidelines] **according to the** [insert name of recommendations, protocol, guidelines] | **I have been able to practice** planning a menu **according to the** Caring for Children guidelines |
| 3. Social/professional role and identity | 9. | [Insert action related to program, intervention, innovation or guidelines] **according to the** [insert name of recommendations, protocol, guidelines], **is part of my role** | Planning a menu **according to the** Caring for Children guidelines, **is part of my role** |
|  | 10. | **It is my responsibility to** [insert action related to program, intervention, innovation or guidelines] **according to the** [insert name of recommendations, protocol, guidelines] | **It is my responsibility to** plan a menu **according to the** Caring for Children guidelines |
|  | 11. | [Insert action related to program, intervention, innovation or guidelines] **according to the** [insert name of recommendations, protocol, guidelines], **is consistent with other aspects of my job** | Planning a menu **according to the** Caring for Children guidelines **is consistent with other aspects of my job** |
| 4. Beliefs about capabilities | 12. | **I am confident that I can** [insert action related to program, intervention, innovation or guidelines] **according to the** [insert name of recommendations, protocol, guidelines] | **I am confident that I can** plan a menu **according to the** Caring for Children guidelines |
|  | 13. | **I am capable of** [insert action related to program, intervention, innovation or guidelines] **according to the** [insert name of recommendations, protocol, guidelines]**,** **even when little time is available** | **I am capable of** planning a menu **according to the** Caring for Children guidelines**,** **even when little time is available** |
|  | 14. | **I have the confidence to** [insert action related to program, intervention, innovation or guidelines] **according to the** [insert name of recommendations, protocol, guidelines]**, even when other professionals I work with are not doing this** | **I have the confidence to** Plan a menu **according to the** Caring for Children guidelines **even when other professionals I work with are not doing this**  **Add interviewer note that ‘other professionals’ refers to the educators that work within the childcare centre** |
|  | 15. | **I have the confidence to** [insert action related to program, intervention, innovation or guidelines] **according to the** [insert name of recommendations, protocol, guidelines]**, even when** [participants, clients, patients, individuals, children] **are not receptive** | **I have the confidence to** plan a menu **according to the** Caring for Children guidelines **even when** the children who attend the service **are not receptive** |
|  | 16. | **I have personal control over** [insert action related to program, intervention, innovation or guidelines] **according to the** [insert name of recommendations, protocol, guidelines] | **I have personal control over** planning a menu **according to the** Caring for Children guidelines |
|  | 17. | **For me,** [insert action related to program, intervention, innovation or guidelines] **according to the** [insert name of recommendations, protocol, guidelines]**, is easy** | **For me,** planning a menu **according to the** Caring for Children guidelines, **is easy** |
| 5. Optimism | 18. | **Even when I feel uncertain about my ability to** [insert action related to program, intervention, innovation or guidelines] **according to the** [insert name of recommendations, protocol, guidelines]**, I usually expect that things will work out okay** | **In uncertain times, when** planning a menu **according to the** Caring for Children guidelines, **I usually expect that things will work out okay**  **Interviewer note:**  Add in examples of uncertain times eg. having new children start at the beginning of the year and not knowing their needs/likes, food budget uncertainty, not knowing how children and staff will respond to new menus |
|  | 19. | **When I** [insert action related to program, intervention, innovation or guidelines] **according to the** [insert name of recommendations, protocol, guidelines]**, I feel optimistic about my job in the future** | **When I** plan a menu **according to the** Caring for Children guidelines**, I feel optimistic about my job in the future** |
|  | 20. | **I do not expect anything will prevent me from** [insert action related to program, intervention, innovation or guidelines] **according to the** [insert name of recommendations, protocol, guidelines] | **I do not expect anything will prevent me from** planning a menu **according to the** Caring for Children guidelines |
| 6. Beliefs about consequences | 21 | **I believe** [insert action related to program, intervention, innovation or guidelines] **according to the** [insert name of recommendations, protocol, guidelines]**, will lead to benefits for the** [participants, clients, patients, individuals, children] | **I believe** planning a menu **according to the** Caring for Children guidelines **will lead to benefits for the** children who attend the service |
|  | 22 | **I believe** [insert action related to program, intervention, innovation or guidelines] **according to the** [insert name of recommendations, protocol, guidelines], **will benefit public health.** | **I believe** planning a menu **according to the** Caring for Children guidelines, **will benefit public health.**  **Interviewer note: define public health ‘Ie. health of the whole population, obesity prevention’** |
|  | 23 | **In my view,** [insert action related to program, intervention, innovation or guidelines] **according to** the [insert name of recommendations, protocol, guidelines], **is practical.** | **In my view,** planning a menu **according to** the Caring for Children guidelines, **is useful** |
|  | 24 | **In my view,** [insert action related to program, intervention, innovation or guidelines] **according to** the [insert name of recommendations, protocol, guidelines], **is worthwhile** | **In my view,** planning a menu **according to** the Caring for Children guidelines **is worthwhile** |
| 7. Reinforcement | 25 | **I get recognition from management at the organisation where I work, when I** [insert action related to program, intervention, innovation or guidelines] **according to the** [insert name of recommendations, protocol, guidelines] | **I get recognition from management at the organisation where I work, when I** plan a menu **according to the** Caring for Children guidelines |
|  | 26 | **When I** [insert action related to program, intervention, innovation or guidelines] **according to the** [insert name of recommendations, protocol, guidelines]**, I get recognition from my colleagues** | **When I** plan a menu **according to the** Caring for Children guidelines **I get recognition from my colleagues** |
|  | 27 | **When I** [insert action related to program, intervention, innovation or guidelines] **according to the** [insert name of recommendations, protocol, guidelines]**, I get recognition from those who it impacts** | **When I** plan a menu **according to the** Caring for Children guidelines**, I get recognition from those who it impacts** |
|  | 28 | **When I** [insert action related to program, intervention, innovation or guidelines] **according to the** [insert name of recommendations, protocol, guidelines]**, I get recognition from** [my Local Government or external agencies] | **When I** plan a menu **according to the** Caring for Children guidelines**, I get recognition from**  Family and Community Services |
| 8. Intentions | 29 | **I intend to** [insert action related to program, intervention, innovation or guidelines] **according to** the [insert name of recommendations, protocol, guidelines], **at** [each/every time relevant to action] | **I intend to** plan a menu **according to** the Caring for Children guidelines **at** every menu review |
|  | 30 | **I will definitely** [insert action related to program, intervention, innovation or guidelines] **according to** the [insert name of recommendations, protocol, guidelines], **at** [each/every time relevant to action] | **I will definitely** plan a menu **according to** the Caring for Children guidelines, **at** every menu review |
|  | 31 | **I intend to** [insert action related to program, intervention, innovation or guidelines] **according to** the [insert name of recommendations, protocol, guidelines], **in the next six months** | **I intend to** plan a menu **according to** the Caring for Children guidelines **in the next six months** |
|  | 32 | **I have a strong intention to** [insert action related to program, intervention, innovation or guidelines] **according to** the [insert name of recommendations, protocol, guidelines], **at** [each/every time relevant to action] | **I have a strong intention to** plan a menu **according to** the Caring for Children guidelines, **at** every menu review |
| 9. Goals | 33 | **Compared to my other tasks,** [insert action related to program, intervention, innovation or guidelines] **according to the** [insert name of recommendations, protocol, guidelines], **is a higher priority on my agenda** | **Compared to my other tasks,** planning a menu **according to the** Caring for Children guidelines **is a higher priority on my agenda** |
|  | 34 | **Compared to my other tasks,** [insert action related to program, intervention, innovation or guidelines] **according to the** [insert name of recommendations, protocol, guidelines], **is an urgent item on my agenda** | **Compared to my other tasks,** planning a menu **according to the** Caring for Children guidelines **is an urgent item on my agenda** |
|  | 35 | **I set achievable short-term goals when** [insert action related to program, intervention, innovation or guidelines] **according to the** [insert name of recommendations, protocol, guidelines] | **I set achievable short-term goals when** planning a menu **according to the** Caring for Children guidelines |
|  | 36 | **I have clear long-term goals related to** [insert action related to program, intervention, innovation or guidelines] **according to the** [insert name of recommendations, protocol, guidelines] | **I have clear long-term goals related to** planning a menu **according to the** Caring for Children guidelines |
| 10. Memory, attention and decision processes | 37 | [Insert action related to program, intervention, innovation or guidelines] **according to the** [insert name of recommendations, protocol, guidelines] **is something I do automatically** | Planning a menu **according to the** Caring for Children guidelines **is something I do automatically** |
|  | 38 | **I can maintain my full attention when I** [insert action related to program, intervention, innovation or guidelines] **according to the** [insert name of recommendations, protocol, guidelines] | **I can maintain my full attention when I** Plan a menu **according to the** Caring for Children guidelines |
|  | 39 | [Insert action related to program, intervention, innovation or guidelines] **according to the** [insert name of recommendations, protocol, guidelines] **is something I forget** | Planning a menu **according to the** Caring for Children guidelines **is something I forget** |
| 11. Environmental context and resources | 40 | **In the organisation I work, all necessary resources are available to** [Insert action related to program, intervention, innovation or guidelines] **according to the** [insert name of recommendations, protocol, guidelines]] | **In the organisation I work, all necessary resources are available to** Plan a menu **according to the** Caring for Children guidelines |
|  | 41 | **I have support from the management of the organisation to** [Insert action related to program, intervention, innovation or guidelines] **according to the** [insert name of recommendations, protocol, guidelines] | **I have support from the management of the organisation to** plan a menu **according to the** Caring for Children guidelines  **Add interviewer note: Specify that management is the nominated supervisor of the service** |
|  | 42 | **The management of the organisation I work for are willing to listen to any problems I have when** [Insert action related to program, intervention, innovation or guidelines] **according to the** [insert name of recommendations, protocol, guidelines] | **The management of the organisation I work for are willing to listen to any problems I have when** planning a menu **according to the** Caring for Children guidelines |
|  | 43 | **The organisation I work for provides the opportunity for training to** [Insert action related to program, intervention, innovation or guidelines] **according to the** [insert name of recommendations, protocol, guidelines] | **The organisation I work for provides the opportunity for training to** plan a menu **according to the** Caring for Children guidelines |
|  | 44 | **The organisation I work for provides sufficient time for me to** [Insert action related to program, intervention, innovation or guidelines] **according to the** [insert name of recommendations, protocol, guidelines] | **The organisation I work for provides sufficient time for me to** plan a menu **according to the** Caring for Children guidelines |
|  | 45 | **The organisation I work for provides sufficient financial support for me to** [Insert action related to program, intervention, innovation or guidelines] **according to the** [insert name of recommendations, protocol, guidelines] | **The organisation I work for provides sufficient financial support for me to** plan a menu **according to the** Caring for Children guidelines |
|  | 46 | [Insert action related to program, intervention, innovation or guidelines]] **according to the** [recommendations, protocol, guidelines] **is included in my organisations reporting outcomes.** | Planning a menu **according to the** Caring for Children guidelines **is included in my organisations** **reporting outcomes.** |
| 12. Social influences | 47 | **People who are important to me think that I should** [Insert action related to program, intervention, innovation or guidelines] **according to the** [insert name of recommendations, protocol, guidelines] | **People who are important to me think that I should** plan a menu **according to the** Caring for Children guidelines |
|  | 48 | **People whose opinion I value would approve of me** [Insert action related to program, intervention, innovation or guidelines] **according to the** [insert name of recommendations, protocol, guidelines] **at** [each/every time relevant to action] | **People whose opinion I value would approve of me** planning a menu **according to the** Caring for Children guidelines **at** every menu review |
|  | 49 | **I can count on support from colleagues whom I work with when things get tough** [Insert action related to program, intervention, innovation or guidelines] **according to the** [insert name of recommendations, protocol, guidelines] **at** [each/every time relevant to action] | **I can count on support from colleagues whom I work with when things get tough** Planning a menu **according to the** Caring for Children guidelines **at** every menu review |
|  | 50 | **Colleagues whom I work with are willing to listen to my problems when** [Insert action related to program, intervention, innovation or guidelines] **according to the** [insert name of recommendations, protocol, guidelines] **at** [each/every time relevant to action] | **Colleagues whom I work with are willing to listen to my problems when** Planning a menu **according to the** Caring for Children guidelines **at** every menu review |
| 13. Emotion | 51 | **I am able to** [insert action related to program, intervention, innovation or guidelines] **according to the** [insert name of recommendations, protocol, guidelines], **in a calm way** | **I am able to** plan a menu **according to the** Caring for Children guidelines, **in a calm way** |
|  | 52 | **I am able to** [insert action related to program, intervention, innovation or guidelines] **according to the** [insert name of recommendations, protocol, guidelines], **in a relaxed way** | **I am able to** plan a menu **according to the** Caring for Children guidelines, **in a relaxed way** |
|  | 53 | **I am able to** [insert action related to program, intervention, innovation or guidelines] **according to the** [insert name of recommendations, protocol, guidelines], **without feeling nervous or anxious** | **I am able to** plan a menu **according to the** Caring for Children guidelines **without feeling anxious** |
|  | 54 | **I am able to** [insert action related to program, intervention, innovation or guidelines] **according to the** [insert name of recommendations, protocol, guidelines], **without feeling distressed or upset** | **I am able to** plan a menu **according to the** Caring for Children guidelines **without feeling distressed or upset** |
|  | 55 | **I am able to** [insert action related to program, intervention, innovation or guidelines] **according to the** [insert name of recommendations, protocol, guidelines], **even when I feel stressed** | **I am able to** plan a menu **according to the** Caring for Children guidelines, **even when I feel stressed** |
| 14. Behavioural regulation | 56 | **I have a detailed plan of how I will** [insert action related to program, intervention, innovation or guidelines] **according to the** [insert name of recommendations, protocol, guidelines] | **I have a detailed plan of how I will** plan a menu **according to the** Caring for Children guidelines, |
|  | 57 | **I have a detailed plan of when I will** [insert action related to program, intervention, innovation or guidelines] **according to the** [insert name of recommendations, protocol, guidelines] | **I have a detailed plan of when I will** plan a menu **according to the** Caring for Children guidelines, |
|  | 58 | **I have a detailed plan on how to** [insert action related to program, intervention, innovation or guidelines] **according to the** [insert name of recommendations, protocol, guidelines] **when** [participants, clients, patients, individuals, children] **are not receptive** | **I have a detailed plan on how to** plan a menu **according to the** Caring for Children guidelines **when** children who attend the service **are not receptive** |
|  | 59 | **I have a detailed plan on how to** [insert action related to program, intervention, innovation or guidelines] **according to the** [insert name of recommendations, protocol, guidelines] **when there is little time** | **I have a detailed plan on how to** plan a menu **according to the** Caring for Children guidelines, **when there is little time** |
|  | 60 | **It is possible to adapt how I** [insert action related to program, intervention, innovation or guidelines] **according to the** [insert name of recommendations, protocol, guidelines] **to meet the my needs as a** [insert role] | **It is possible to adapt how I** plan a menu **according to the** Caring for Children guidelines **to meet the my needs as a** service cook |
|  | 61 | [Insert action related to program, intervention, innovation or guidelines] **according to the** [insert name of recommendations, protocol, guidelines] **is compatible with other aspects of my job** | Planning a menu **according to the** Caring for Children guidelines **is compatible with other aspects of my job** |
